# Supplementary material for: Detection and imaging of chemicals and hidden explosives using terahertz time-domain spectroscopy and deep learning
Source: Light Sci Appl. 2026 Jan 22;15:80. doi: 10.1038/s41377-026-02190-z (PMC12824377; doi:10.1038/s41377-026-02190-z)
Supplement: Supplementary file 1 — Supplementary materials for Detection and imaging of chemicals and hidden explosives using terahertz time-domain spectroscopy and deep learning [file 41377_2026_2190_MOESM1_ESM.pdf]

Supplementary Information for

Detection and imaging of chemicals and hidden explosives using terahertz time-domain spectroscopy and deep learning

Xinghe Jiang[1, 2, +] email: xinghej@g.ucla.edu

Yuhang Li[1, 2, 3, +] email: yuhangli@g.ucla.edu

Yuzhu Li[1, 2, 3] email: liyuzhu@g.ucla.edu

Che-Yung Shen[1, 2, 3] email: steven121200@g.ucla.edu

Aydogan Ozcan[1, 2, 3, *] email: ozcan@g.ucla.edu

Mona Jarrahi[1, 2, *] email: mjarrahi@ucla.edu

[1]Electrical and Computer Engineering Department, University of California, Los Angeles, California 90095, USA

[2]California NanoSystems Institute (CNSI), University of California, Los Angeles, California 90095, USA

[3]Bioengineering Department, University of California, Los Angeles, California 90095, USA

[+]Equal contributing authors

*Correspondence:

Mona Jarrahi E-mail: mjarrahi@ucla.edu

Aydogan Ozcan E-mail: ozcan@g.ucla.edu

Address: 420 Westwood Plaza, Eng. IV 66-147E, UCLA, Los Angeles, CA 90095, USA

Phone: +1 (310) 206-1371

Fax: +1 (310) 825-8282

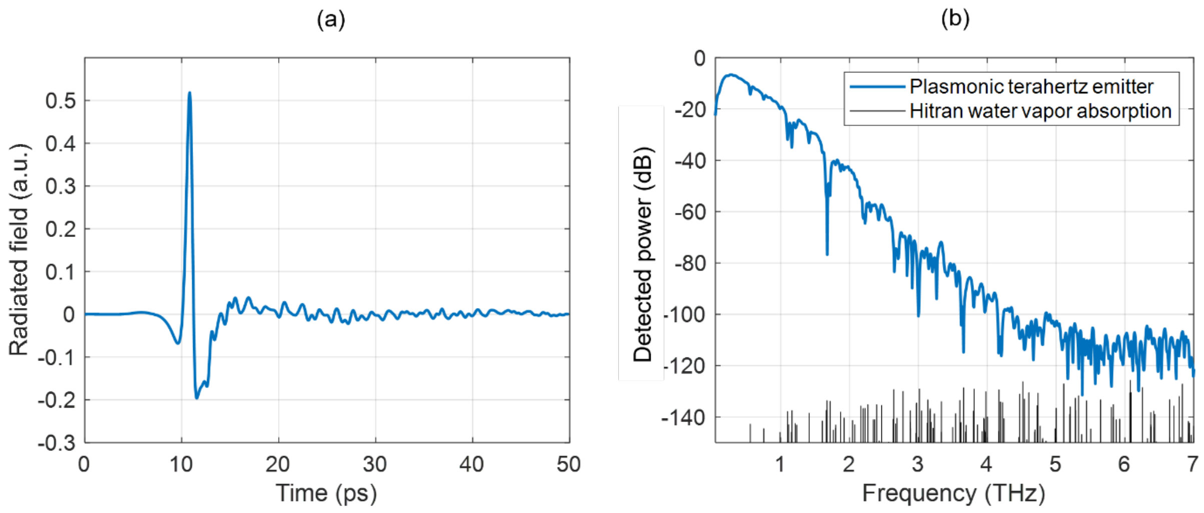

Figure S1. (a) Terahertz time-domain response of the developed THz-TDS system in transmission mode. (b) Corresponding power spectrum. To benchmark against state-of-the-art systems—typically characterized in transmission mode—we evaluated our system in transmission mode. The system achieves a peak dynamic range of 104 dB and a bandwidth of 5 THz for a 50 ps scan range and a 3-second scan time. Note that in reflection mode, the use of a 50:50 terahertz beam splitter introduces at least a 6 dB reduction in signal-to-noise ratio (SNR).

THz-TDS system	Dynamic range (dB)	Bandwidth (THz)	Measurement time (s)	Scan range (ps)
THz-TDS used here	104	5	3	50
Menlo Tera K15[1]	110	6	1.5	50
Menlo TeraSmart[2]	110	6	1.5	50
TeraFlash pro[3]	95	6	20	50
TeraFlash smart[4]	80	4.5	60	150
Advantest TAS7500TS[5]	70	4	262	131

Table S1. Performance comparison of our THz-TDS system in transmission mode with the state-of-the-art systems.

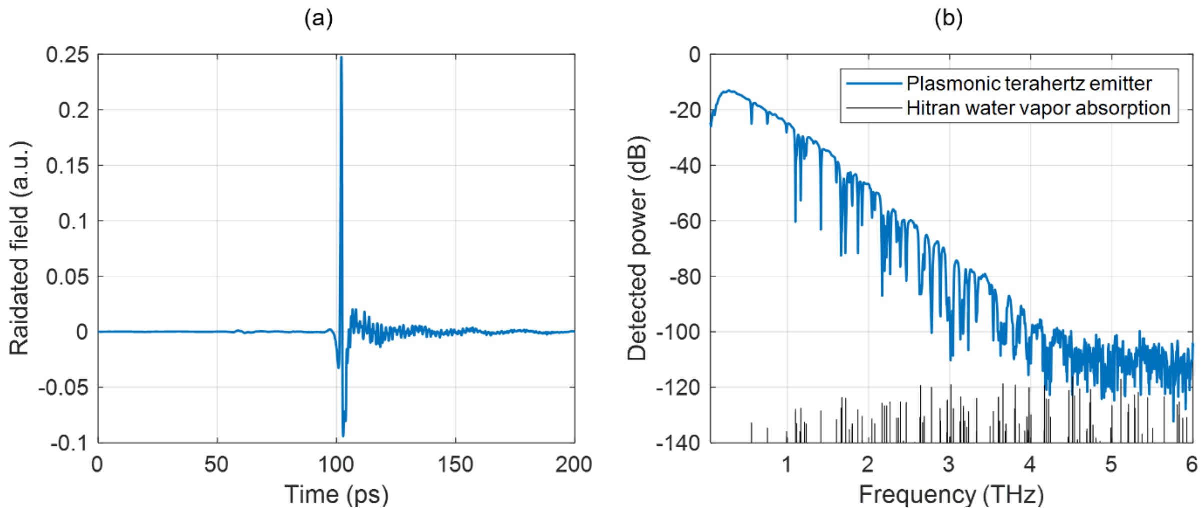

Figure S2. (a) Terahertz time-domain response of the system without a sample on the copper platform. (b) Corresponding terahertz power spectrum. The system achieves a peak dynamic range of 96 dB and a bandwidth of 4.5 THz in a measurement time of 3 seconds.

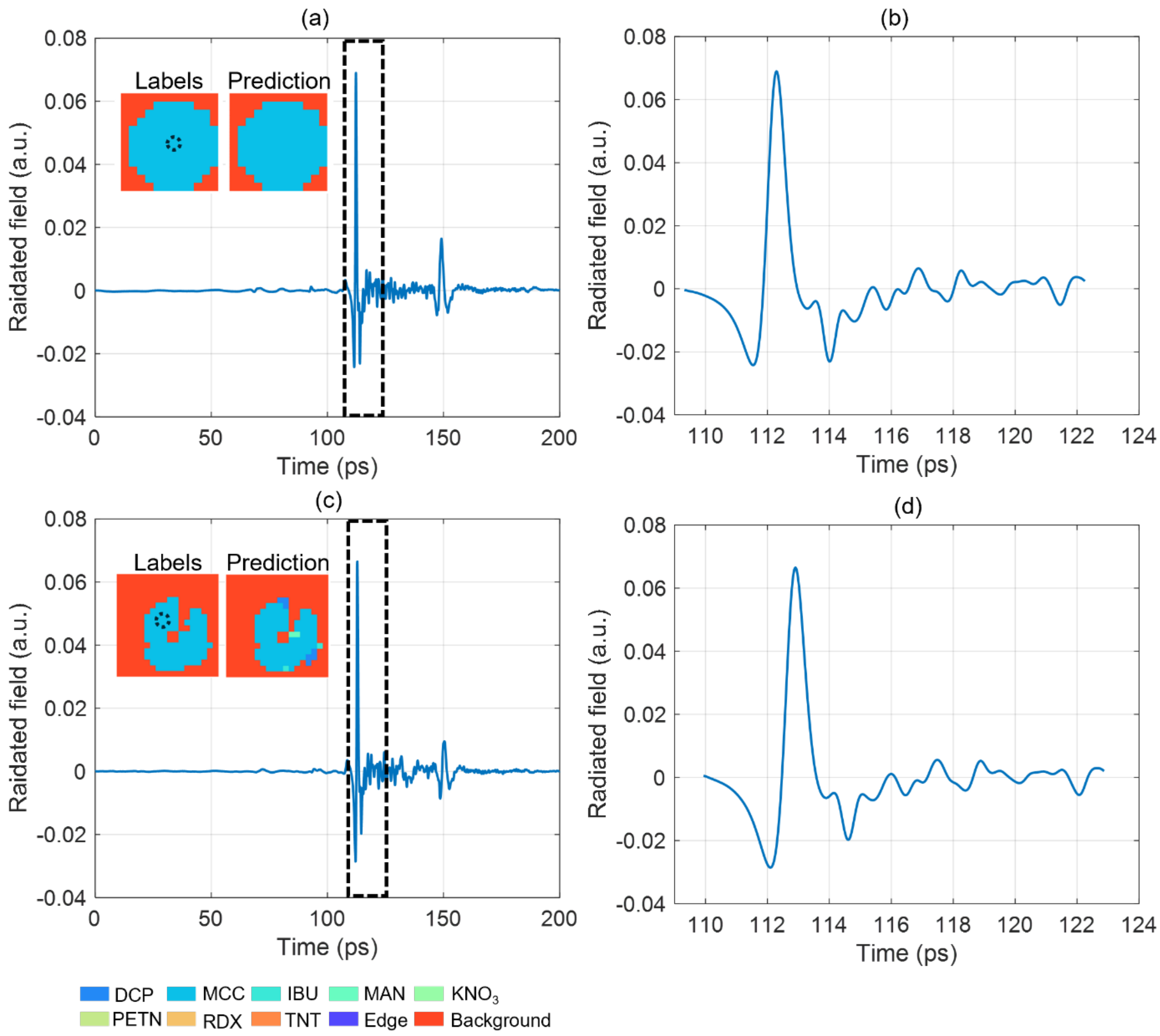

Figure S3. Terahertz time-domain responses of MCC. (a) The full time-domain response of an intact MCC tablet at one pixel. Inset figures: the scanned pixel in the labeled ground truth and the prediction of the network. (b) One of the separated pulses from (a), which is P3, i.e., the pulse reflected from the bottom of the tablet. (c) The full time-domain response of a cracked MCC tablet at one pixel. Inset figures: the scanned pixel in the labeled ground truth and the prediction of the network. (d) One of the separated pulses from (c), which is P3, i.e., the pulse reflected from the bottom of the tablet. The time delay and relative amplitude between two pulses are different in (a) and (c). The separated pulses in (b) and (d) are similar, enabling accurate pulse-based predictions.

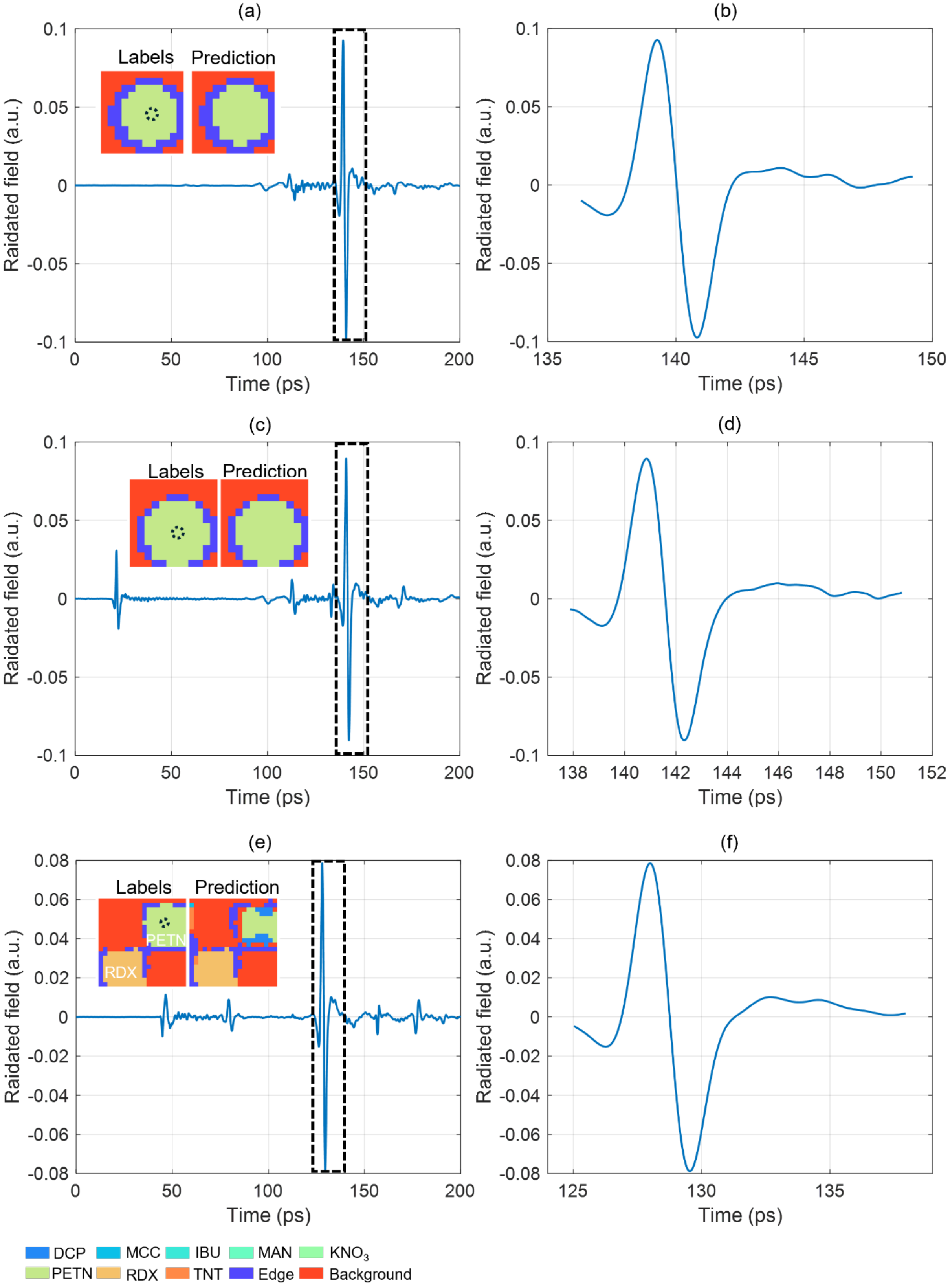

(a)
(b)
(c)
(d)
(e)
(f)
Labels
Prediction
PETN
RDX
Raidated field (a.u.)
Radiated field (a.u.)
Time (ps)
DCP
MCC
IBU
MAN
KNO3
PETN
RDX
TNT
Edge
Background

Figure S4. Terahertz time-domain responses of PETN. (a) The full time-domain response of PETN at one pixel. Inset figures: the scanned pixel in the labeled ground truth and the prediction of the network. (b) One of the separated pulses from (a), which is P3, i.e., the pulse reflected from the bottom of PETN. (c) The full time-domain response of PETN at one pixel under paper cover. The first pulse in the trace is the reflection from the paper cover. Inset figures: the scanned pixel in the labeled ground truth and the prediction of the network. (d) One of the separated pulses from (c), which is P3, i.e., the pulse reflected from the bottom of PETN. (e) The full time-domain response of PETN at one pixel under paper cover with RDX in the same FOV. (f) One of the separated pulses from (e). Although (a), (c), (e) are different because of geometric differences, the separated pulses (b), (d), (f) are similar. The neural network analyzed all the pulses in (a), (c), (e) individually without knowing whether the pulse comes from the paper cover or the sample, achieving accurate detection.

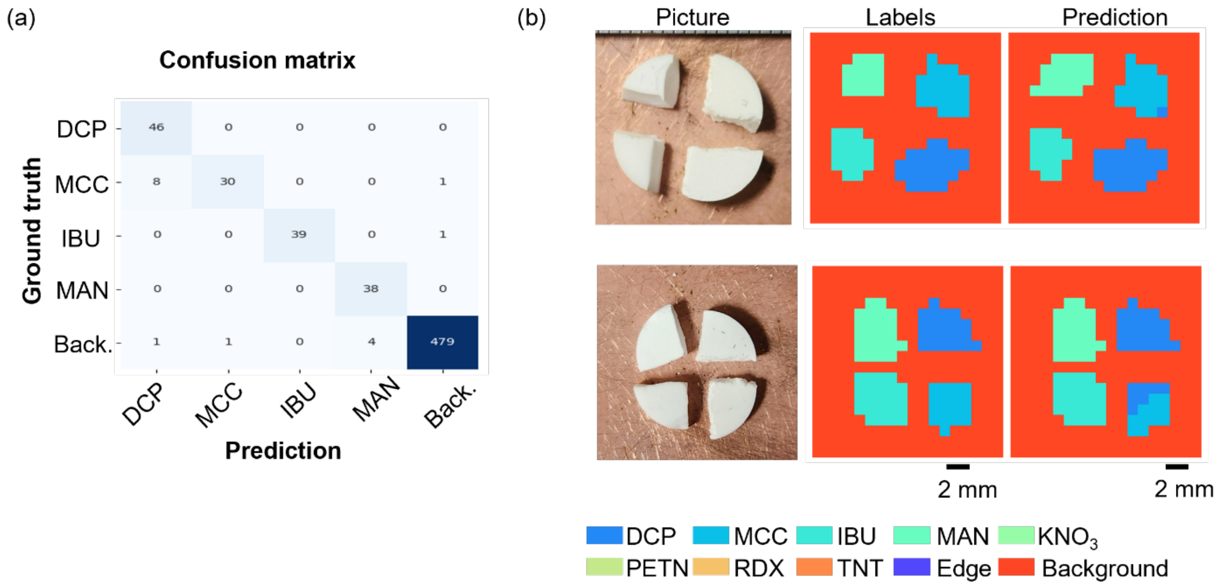

Figure S5. Classification results for four different types of cracked pharmaceutical samples in the same field of view. (a) Confusion matrix summarizing the classification performance. The classification accuracy for DCP, MCC, IBU, MAN, and background are 100%, 82.86%, 100%, 100%, 98.76%, respectively. The average accuracy is 98.13%. (b) Pictures of the cracked samples, ground truth labels and our network predictions.

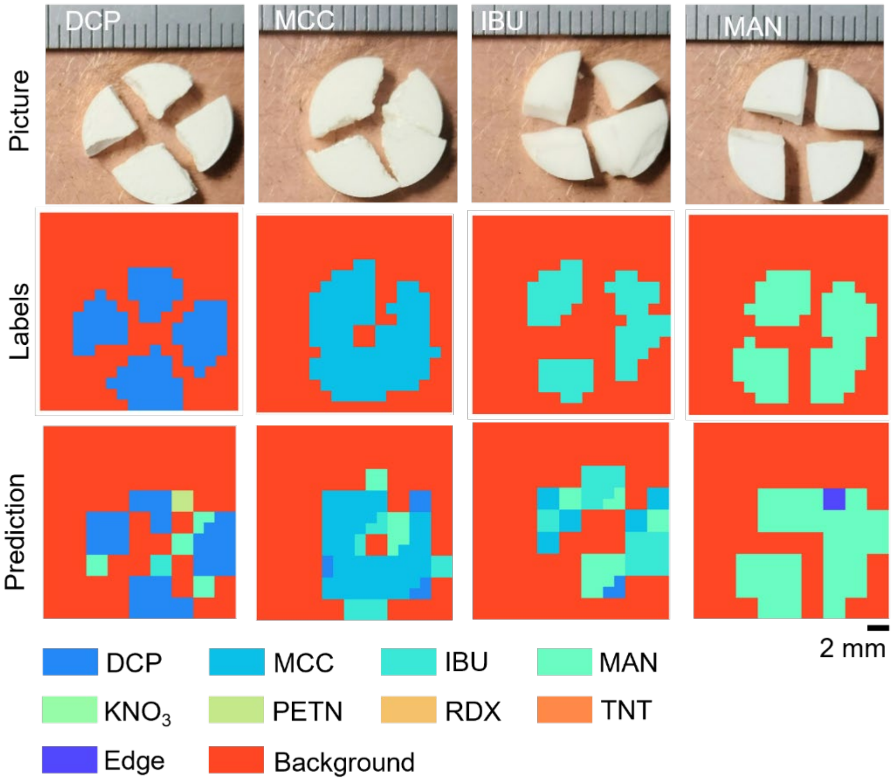

Figure S6. Classification results for cracked pharmaceutical samples with a step size of 2 mm. To investigate the effect of spatial resolution, we down-sampled the dataset for the cracked samples (Fig. 4c) to simulate a coarser 2-mm step size. Compared to the 1-mm results, this led to a significant degradation in performance, with prediction accuracies dropping to 67.61% for DCP, 60.93% for MCC, 51.30% for IBU, 82.01% for MAN, and 91.43% for the background. The average accuracy is 84.14%. These results underscore that fine spatial resolution is crucial for accurately classifying irregularly shaped chemical samples. A higher resolution provides more pixels for a given area, which enhances the reliability of the majority voting process and, in turn, boosts the final prediction accuracy.

References

1. Menlo Systems GmbH. https://www.menlosystems.com/products/thz-time-domain-solutions/terak15-terahertz-spectrometer/ (2025).
2. Menlo Systems GmbH. https://www.menlosystems.com/products/thz-time-domain-solutions/terasmart-terahertz-spectrometer/ (2025).
3. TOPTICA Photonics AG. https://www.toptica.com/products/terahertz-systems/time-domain/teraflash-pro (2025).
4. TOPTICA Photonics AG. https://www.toptica.com/products/terahertz-systems/time-domain/teraflash-smart (2025).
5. ADVANTEST CORPORATION. https://www.advantest.com/en/products/scientific-equipment-medical-systems/terahertz-imaging/tas7500/ (2025).
